# Cocktail Strategy Based on NK Cell-Derived Exosomes and Their Biomimetic Nanoparticles for Dual Tumor Therapy

**DOI:** 10.3390/cancers11101560

**Published:** 2019-10-14

**Authors:** Guosheng Wang, Weilei Hu, Haiqiong Chen, Xin Shou, Tingting Ye, Yibing Xu

**Affiliations:** Institute of Translational Medicine, Zhejiang University School of Medicine, Hangzhou 310029, China; 21518460@zju.edu.cn (G.W.); weileihu@zju.edu.cn (W.H.); 182594@zju.edu.cn (H.C.); shouxin@zju.edu.cn (X.S.); tingtingye@zju.edu.cn (T.Y.)

**Keywords:** antitumor strategy, biomimetic core–shell nanoparticles, NK cell-derived exosomes, drug delivery system

## Abstract

Successful cancer therapy requires drugs being precisely delivered to tumors. Nanosized drugs have attracted considerable recent attention, but their toxicity and high immunogenicity are important obstacles hampering their clinical translation. Here we report a novel “cocktail therapy” strategy based on excess natural killer cell-derived exosomes (NKEXOs) in combination with their biomimetic core–shell nanoparticles (NNs) for tumor-targeted therapy. The NNs were self- assembled with a dendrimer core loading therapeutic miRNA and a hydrophilic NKEXOs shell. Their successful fabrication was confirmed by transmission electron microscopy (TEM) and confocal laser scanning microscopy (CLSM). The resulting NN/NKEXO cocktail showed highly efficient targeting and therapeutic miRNA delivery to neuroblastoma cells in vivo, as demonstrated by two-photon excited scanning fluorescence imaging (TPEFI) and with an IVIS Spectrum in vivo imaging system (IVIS), leading to dual inhibition of tumor growth. With unique biocompatibility, we propose this NN/NKEXO cocktail as a new avenue for tumor therapy, with potential prospects for clinical applications.

## 1. Introduction

Targeted drug delivery systems for cancer therapy have attracted considerable recent attention owing to their high delivery efficiency along with minimal side effects [1,2,3]. Polymers are of great significance in targeted drug delivery as nanocarriers for biomacromolecular agents. Among them, polyamidoamine (PAMAM), the first reported and commercialized dendrimers, are hyperbranched polymers with a globular structure, which efficiently transfer nucleic acids (DNA, siRNA, miRNA, etc.) to various cell types and are safer than other cationic polymers [4,5,6]. PAMAM dendrimers have a distinctive structural feature with positively charged amino groups present at internal cavities, which can interact with negatively charged nucleic acids to form dendriplexes [7,8]. In order to improve their biocompatibility and targeting ability, many strategies have been taken to modify the PAMAM surface, including peptide conjugation, carbohydrate conjugation, acetylation and PEGylation [9].

More recently, biomembrane camouflage strategies have been investigated in cancer therapy. This strategy refers to nanocarriers coated with natural biomembrane components, making them highly versatile in cargo encapsulation and surface functionalization. Many types of membranes have been used to fabricate membrane-camouflaged nanoparticles, including membranes from red blood cells, platelets, white blood cells, cancer cells and bacteria [10,11,12,13,14,15]. Meanwhile, incorporation of membrane proteins within the bilayer of biomimetic nanovesicles using a microfluidic-based platform offers a one-step solution to endow nanoparticles with multiple biological feature such as evading the mononuclear phagocytic system, crossing the biological barriers or targeting inflamed tissues [16,17,18]. However, without exception, fabrication of core–shell nanoparticles has required extraction of plasma membrane materials by cell lysis and membrane purification, a process that greatly increases the risk of contamination and destruction of functional surface proteins. Therefore, it is of paramount importance for their clinical application to obtain functional off-the-shelf and highly biocompatible materials and establish an efficient and handy synthetic process [19].

Natural killer (NK) cells, known to play a critical role in tumor immunology [20,21] secret exosomes expressing killer proteins, including FASL and perforin, cytotoxic to multiple tumors [22,23,24,25]. Previous studies have also demonstrated that natural killer cell-derived exosomes (NKEXOs) have tumor-specific accumulation with no cytotoxic activity against normal tissues [22,23]. Meanwhile, acidic tumor microenvironment facilitates the accumulation of this nanoparticles [26]. Hence, NKEXOs can perform a dual function: They can facilitate tumor targeting and act as direct antitumor agent. More importantly, NK cell-based therapies can act as a renewable product in an allogeneic setting and be given to patients without causing graft-versus-host disease [27]. For these advantages, we ask whether intact NKEXOs could be used directly as camouflage material to greatly simplify synthetic steps and avoid contamination of immunogenic substances due to the destruction progress of membrane extraction.

## 2. Results

### 2.1. Characterization and Function of Isolated NKEXOs

**Figure 1:**
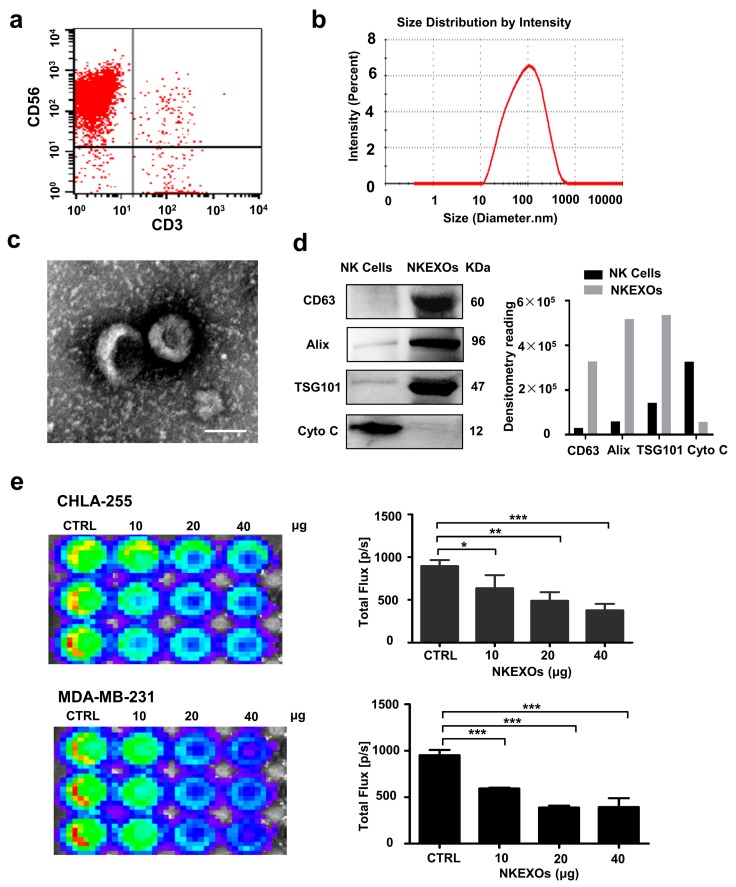
Characterization and function of isolated NKEXOs. (**a**) Flow cytometric analysis of NK cells (CD3− and CD56+) grown for a total of 21 days. (**b**) Size distributions of NKEXO measured by dynamic light scattering show peak diameters at 100 nm. (**c**) The morphology of exosomes was analyzed by transmission electron microscopy. (**d**) Western blot analysis of CD63, ALIX, TSG101 and cytochrome c (Cyto C) expression level in NK cells and NKEXOs. (**e**) MDA-MB-231luc and CHLA-255luc cell lines (10^4^ cells per well) were incubated with control medium and different amounts of NKEXOs (10, 20 and 40 µg) for 24 h. Cell viability was assessed by bioluminescence imaging. Each bar represents the mean ± SD of three replicates. * p < 0.05; ** p < 0.01 and *** p < 0.001.

To prepare the nanoparticle (NN)/NKEXO cocktail, we expanded the NK cells to a 94% purity level by co-culturing peripheral blood mononuclear cells with artificial antigen-presenting cells (aAPC) as previously reported (Figure 1a) [25,28]. Then, NKEXOs were isolated and purified from the culture supernatants of NK cells by differential centrifugation [29]. Their size was further measured by dynamic light scattering, showing a mean particle diameter of 100 nm (Figure 1b). In the purified extracts, typical exosome structures were observed by transmission electron microscopy TEM (Figure 1c). Western blot (WB) analysis indicated that NKEXOs contain typical exosomal proteins (Alix, TSG101 and CD63), together with the absence of cytochrome C (Figure 1d). These data are consistent with those previously reported for typical exosomes [30]. To verify their cytotoxicity towards tumor cells, MDA-MB-231-luc and CHLA-255-luc cells were incubated with NKEXOs for 24 h. As reported previously [22,23,24,25], cell viability decreased in both tumor cell lines in a dose-dependent manner (Figure 1e). Internalization of PKH26-labeled NKEXOs into tumor cells was also observed with confocal laser scanning microscopy (CLSM) with a rotating 3D rendering of z-stack images (Figure S1; Video S1).

### 2.2. Fabrication and Characterization of the NN/NKEXO Cocktail

**Figure 2:**
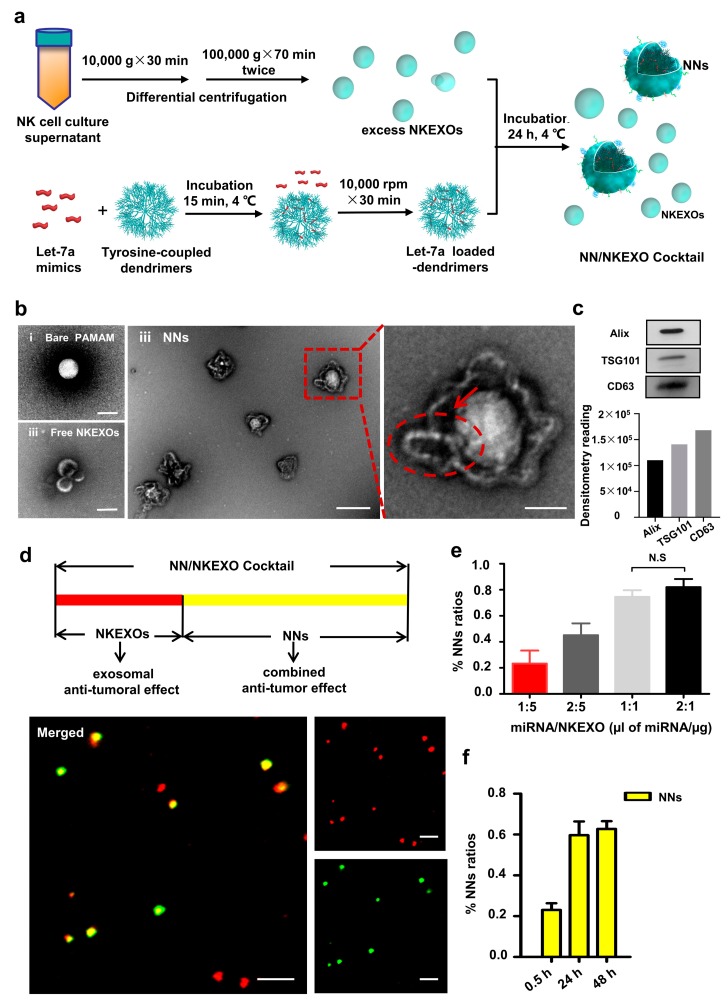
Fabrication and characterization of the NN/NKEXO cocktail. (**a**) Schematic design of the NN/NKEXO cocktail. (**b**) TEM images of bare PAMAM dendrimers (i), NNs (ii) and free NKEXO in supernatant (iii). The arrow indicates an integrated NKEXO. (**c**) NNs contain typical intracellular proteins markers (ALIX, TSG101), confirmed by Western blot (WB). (**d**) CLSM images of the fabricated NN/NKEXO cocktail illustrating the colocalization of let-7a loaded dendrimers (green) and NKEXO (red). (**e**) Percentages of NNs in the NN/NKEXO cocktail at different miRNA to NKEXO ratios. (**f**) Percentages of NNs in NNs-NKEXO cocktail at different synthesis times at a 1:1 miRNA to NKEXO ratio.

PAMAM dendrimer have been used previously for nucleic acid delivery [4,5,6]. Using the intrinsic hydrophobicity of the lipid bilayers in the exosome, tyrosine was linked to the dendrimers to promote the binding process [31]. Meanwhile, the phenyl groups of the tyrosine are able to disrupt the endosomal membrane, facilitating release of the therapeutic miRNA [32,33]. The NN/NKEXO cocktail was then prepared by simple incubation of the purified NKEXOs with tyrosine-coupled dendrimers preloaded with let-7a mimics (Figure 2a). The resulting NNs were further characterized by TEM, displaying a core–shell structure indicating the presence of the dendrimers in the core and NKEXOs in the shell (Figure 2b). More importantly, the precipitated NNs contained typical intracellular proteins including TSG101 and Alix, indicating the shell was made of intact exosomes (Figure 2c). Furthermore, the successful coating of the NKEXOs was confirmed by colocalization of the PKH26 (red)-labeled NKEXOs and Fluorescein phosphoramidites FAM (green)-labeled dendrimers (Figure 2d).

In order for the dendrimer core to be fully encapsulated, it is necessary to ensure that the exosomes are always in an excessive state. As an easy and reliable quality control, we used a high-content screening (HCS) system (Operetta, PerkinElmer, Massachusetts, USA) combined with fluorescent staining to determine the parameters for the cocktail synthesis. The module and parameter settings of the instrument were set as shown in Table S1. In each quality control process, we first fixed the amount of exosomes and used different amounts of let-7a mimics. Thus, the amount of let-7a mimics corresponding to the highest NNs synthesis rate was the maximum amount that could be loaded, and this amount or below was optional.

As shown in Figure 2e, for instance, we used fixed amounts of NKEXOs 10 μg and varied the amount of let-7a mimics and the synthesis time. Using HCS screening, we observed that the coupling efficiency of NNs increased over time and reached a peak of nearly 59.67% (±6.69%) at 24 h. In contrast, the highest NNs synthetic rate was observed when the amount of the let-7a mimics was 0.13 μg (10 μL at 1 μM concentration; Figure 2f). This was considered the maximum rate to prepare a single batch of the NN/NKEXO cocktail over a 24 h period. All the cocktails used in this study were subject to the same progress to ensure that NKEXOs are excess at any time.

### 2.3. Binding and Cytotoxicity of the NN/NKEXO Cocktail to a Human Neuroblastoma Cell Line In Vitro

**Figure 3:**
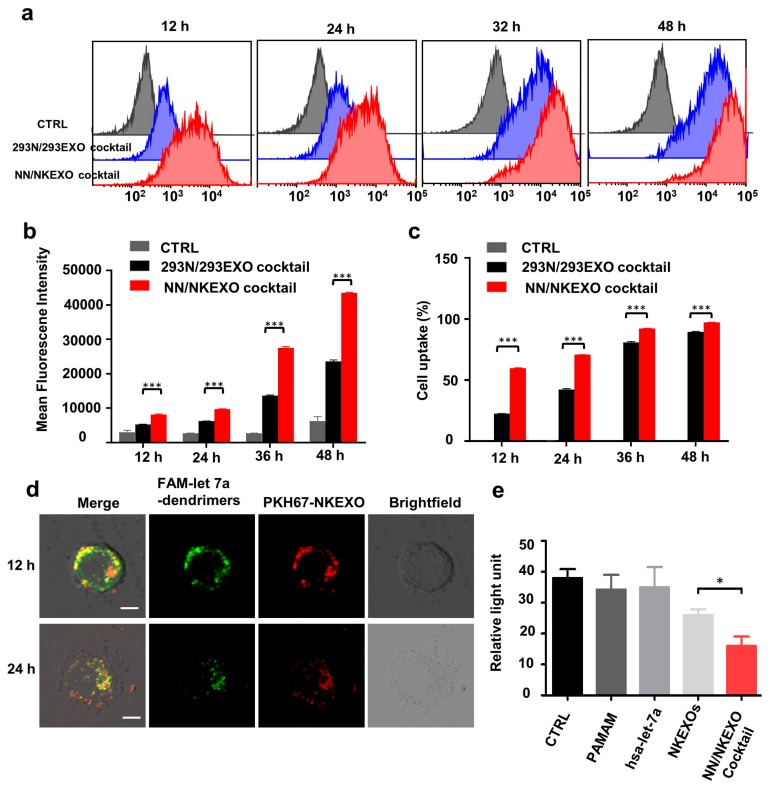
Binding and cytotoxicity of the NN/NKEXO cocktail to a human neuroblastoma cell line in vitro. (**a**) Representative confocal fluorescence images of CHLA-255 cells after treatment with NN/NKEXO cocktail for 12 and 24 h (let-7a-dendrimer: Green; NKEXO: Red). (**b**) Quantitative flow cytometric analysis of CHLA-255 cells incubated with control medium, 293N-293Exo cocktail and NN/NKEXO cocktail both made of let-7a (0.5 µg) and their exosomes (50 µg) for 12, 24, 36 and 48 h; *n* = 3. (**c**) Mean fluorescence intensity of CHLA-255 cells after 12, 24, 36 or 48 h incubation with control medium and different cocktails; *n* = 3. (**d**) Percentages of cells with increased miRNA fluorescence; *n* = 3. (**e**) Cell viability of CHLA-255 cells (10^4^ cells per well) after treatment with PBS (CTRL), PAMAM, let-7a, NKEXOs and NN/NKEXO cocktail (40 μg NKEXOs and 0.05 μg let-7a) for 24 h. Data are presented as mean ± SD; *n* = 3.

Next, we evaluated the nanoparticle cocktail kinetics in human neuroblastoma CHLA-255 cells at different incubation times with the dual labeled cocktail (miRNA loaded dendrimers: FAM, green and NKEXOs: PKH-26, red), followed by observation via CLSM. As shown in Figure 3a, the internalization of both NKEXOs and let-7a inside tumor cells increased from 12 to 24 h. Additional quantitative measurements were performed using flow cytometry. Equivalent exosomes derived from human embryonic kidney cell line 293T were obtained and 293N/293EXO cocktail was synthesized as control. The fluorescence intensity of let-7a mimics in CHLA-255 cells increased along with the NKEXO fluorescence in a time-dependent manner. Neither fluorescence intensities weakened until cells were incubated for at least 48 h. Importantly, CHLA-255 cells treated with the NN/NKEXO cocktail showed a prominent right shift in the cytometric analysis, suggesting a higher cellular uptake of exosomes than 293N/293EXO cocktail (Figure 3b). Furthermore, more CHLA-255 cells internalized miRNA, with the mean fluorescence intensity value higher than in the control cocktail at any time point (Figure 3c,d). This indicates that the NKEXO based outer shell plays a critical role in improving the cancer target drug delivery efficiency. We also used the bioluminescence assay to substantiate the synergistic apoptosis-inducing capability of the cocktail in vitro. Since let-7a, a therapeutic microRNAs, is closely involved in the proliferation of neuroblastomas [34,35]. As shown in Figure 3e, after 24 h of incubation, NN/NKEXO cocktail-let-7a exhibited significantly higher cytotoxicity compared with NKEXO alone in neuroblastoma cells. In contrast, no significant inhibition of cell growth was observed in let-7a or dendrimers treated groups, indicating that the NNs mediated delivery of therapeutic let-7a enhances the anti-tumor effect of the cocktail by triggering a synergistic induction of apoptosis.

### 2.4. In Vivo Biodistribution and Anti-Tumor Effect of the NN/NKEXO Cocktail

**Figure 4:**
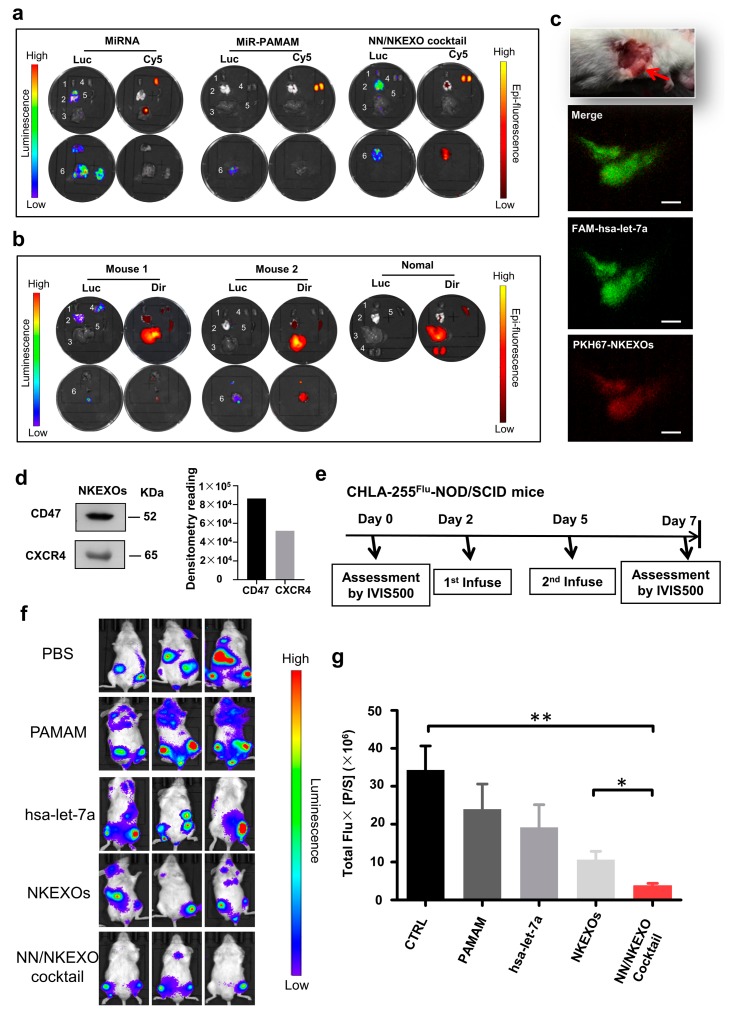
In vivo biodistribution and anti-tumor effect of the NN/NKEXO cocktail. (**a**) Bioluminescence (Luc) and miRNA fluorescence (Cy5) images of major organs of the NOD/SCID mice bearing systemic CHLA-255-luc tumors 6 h after intravenous (i.v.) injection of Cy5-let-7a, Cy5-let-7a-dendrimers and Cy5-labeled NN/NKEXO cocktail: 1) Heart; 2) lung; 3) liver; 4) kidney; 5) spleen and 6) tumor. (**b**) Bioluminescence (Luc) and fluorescence images of major organs 6 h after i.v. injection of DiR-labeled NN/NKEXO cocktail made of 5.28 µg of let-7a and 100 μg of NKEXOs. (**c**) In vivo two-photon excitation fluorescence images of the CHLA-255^luc^ subcutaneous tumor model 20 min after i.v. injection of 400 μL dual fluorescently labeled NN/NKEXO cocktail made of 5.28 µg of let-7a and 100 μg of NKEXOs. (**d**) Expression of functional proteins (CD47 and CXCR4) was confirmed by WB. (**e**) Experimental protocol for CHLA-255^luc^-NOD/SCID mice shown in (**f**). (**f**) Bioluminescence imaging (BLI) performed in CHLA-255^luc^-NOD/SCID mice treated with different cocktail formulations at let-7a dose of 5.28 µg and NKEXO dose of 100 µg (*n* = 3). (**g**) Quantification of BLI results. Each bar represents mean ± SD of three replicates. * p < 0.05 and ** p < 0.001.

To evaluate the tumor targeting capability of the cocktail, let-7a was labeled with Cy5 and the cocktail was administrated intravenously into the CHLA-255-luc tumor-bearing nonobese diabetic/severe combined immunodeficient (NOD/SCID) mice. After removal of the tumors and organs of interest 6 h after injection, ex vivo fluorescence imaging showed strong fluorescence signals of let-7a loaded dendrimers in the tumor tissues and relatively strong signals in the kidneys. In contrast, no specific fluorescence was detected in tumor tissues of mice treated with miRNA alone or miRNA loaded dendrimers (Figure 4a). These results indicate that the NN/NKEXO cocktail have highly efficient targeting and miRNA delivery to tumor cells. NKEXOs showed tumor-specific accumulation as early as 24 h post-injection in vivo [6]. Here, we labeled NKEXOs with DiR (red) to prepare the cocktail administrated intravenously. Interestingly, using ex vivo fluorescence imaging (Figure 4b), NKEXOs showed tumor-specific accumulation as early as 6 h post-injection. In the second method, a subcutaneous tumor mice model was used. The dual fluorescently labeled cocktail (let-7a loaded dendrimers: FAM, green and NKEXOs: PKH-26, red) was intravenously injected. As demonstrated by two-photon excited scanning fluorescence imaging (TPEFI), miRNA loaded dendrimers were found in the tumor tissues as early as 20 min after administration (Figure 4c; Video S2). These results indicate that our cocktails are suitable for highly efficient tumor-targeted delivery. In this regard, CXCR4 presence in NKEXOs has been shown which intimately linked to the accumulation at specific organs, and CD47 presence is a “don’t eat me” signal for phagocytic cells to achieve longer circulation times (Figure 4d) [36,37,38]. Both molecules potentially promoted NNs to target primary tumors and organ specific metastasis more accurately and actively.

Finally, we evaluated the in vivo antitumor effect of the cocktails. Mice bearing tumors induced by CHLA-255-luc cells were randomly sorted into five groups (*n* = 3 per group). As indicated in Figure 4e, a pronounced suppression of tumor growth was observed in the animals treated with cocktail-let-7a. Notably, the signal intensity of the cocktail treatment group was significantly lower than that of the NKEXO group *(p* < 0.05), further indicating that the let-7a was efficiently and successfully carried to tumor cells by NNs and exerted a synergistic antitumor effect with NKEXOs (Figure 4f,g).

## 3. Discussion

An ideal nanomedicine for cancer therapy should elicit minimal immunogenicity. In the traditional membrane preparation processes, the use of integrated NKEXOs as shells of nanoparticles avoids potential contamination with immunogenic substances. The advantage of NK cells, a source of NKEXOs, is that it can act as an off-the-shelf product for allogeneic settings [27]. NKEXOs also exhibit the benefits of being stable vesicles that maintain their biological activities for at least two years at −80 °C [39]. These properties make them more suitable for clinical applications.

Although the exact mechanisms by which NKEXOs specifically target tumors are not clear, the following clues may explain this targeting and guide further research in this area. CXCR4 is one of the best-characterized chemokine receptors mediating leukocyte trafficking [40]. Moreover, its ligand, SDF-1a, is released in large amounts by some tumor or organs, such as the lung and liver [36,37]. The interaction between SDF-1 and CXCR4 also plays an important role in cancer metastasis [41,42]. NKEXOs, expressing CXCR4 as determined in our study, likely induced NNs to leave systemic circulation and traffic into tumors or organs. In this context, NNs had the potential to become “prophylactic” anti-metastatic agents.

However, we note that in our experiments the number of experimental animals was relatively small with a short observation period. Besides, more details of the coating progress from a chemical standpoint were not provided.

Given the distinct advantage of being easy to generate and having good clinical application prospects, we believe further research in this field will resolve the aforementioned limitations. To this end, this cocktail strategy is expected to offer new opportunities for the advancement of cancer nanomedicine.

## 4. Materials and Methods

### 4.1. Cell Culture

The human breast cancer cell lines MDA-MB-231 and human embryonic kidney cell lines 293T were propagated in DMEM (Gibco, Grand Island, NY, USA) supplemented with 10% FBS (Gibco) and 1% penicillin-streptomycin (Hyclone, Logan, UT, USA). Human neuroblastoma cells CHLA-255 were incubated in IMDM (Gibco) with 20% FBS and 1% penicillin-streptomycin. All cell lines were purchased from the American Type Culture Collection (ATCC). Both MDA-MB-231 and CHLA-255 cells were transfected by Nucleofection™ (Lonza, Cologne, Germany) with a firefly luciferase expression plasmid. All cells were maintained in a humidified 5% CO_2_ atmosphere at 37 °C.

### 4.2. Preparation and Function of NK Cell Derived Exosomes

#### 4.2.1. Isolation of Exosomes

We isolated NK cell-derived exosomes using differential ultracentrifugation according to the literature. On day 21, NK cell culture supernatants were harvested after a preliminary centrifugation at 400× g for 10 min to eliminate cells. The supernatants were centrifuged first at 2000× g for 10 min and then at 10,000× g for 70 min to remove dead cells and cell debris, followed by an additional centrifugation step at 100,000× g for 70 min in a 70Ti ultracentrifuge rotor (Optima™XPN-100, Beckman, Brea, CA, USA) to obtain pellets containing raw exosomes. The pellets were then purified by washing them with PBS and centrifuged at 100,000× g for another 70 min. The isolated exosomes were resuspended in PBS (100:1 enrichment) and stored at −80 °C before use. All procedures were carried out at 4 °C. We isolated exosomes from the 293T culture medium in the same way.

#### 4.2.2. Measurement of Particle Size

A dynamic light scattering (DLS) system equipped with a 532-nm laser (Malvern Instruments, Malvern, UK) was used for exosome particle size analysis. Samples were diluted before analysis (10 μL, 1:100 diluted in PBS) and each sample was measured three times. Data was collected and analyzed by Dispersion Technology Software (Malvern Instruments). The size distribution was measured by signal intensity and the Z-average diameter obtained from the autocorrelation function using the general-purpose mode.

#### 4.2.3. Determination of Protein Concentration

Protein concentration in isolated exosomes was calculated by the bicinchoninic acid protein assay. Briefly, 25 µL of an aqueous suspension of isolated exosomes after lysis were added to 200 µL of standard working reagent in wells of a 96-well plate. Absorbance of the mixture was determined at 562 nm with a microplate reader and the protein concentration estimated. A calibration curve was constructed using bovine serum albumin as protein standard.

#### 4.2.4. Western Blotting (WB) Analysis

Presence of marker proteins in exosomes was analyzed and confirmed by WB. Proteins from lysed cells or isolated exosomes were denatured and loaded onto sodium dodecyl sulfate polyacrylamide gels, transferred to polyvinylidene difluoride membranes (Millipore, Billerica, MA, USA), and subsequently stained with the corresponding primary and secondary antibodies. The following antibodies were used for WB analysis according to manufacturer’s instructions: Anti-ALIX polyclonal antibody (12422-1-AP, Proteintech, Rosemont, IL, USA), anti-CD47 polyclonal antibody (ab108415, Abcam, Cambridge, MA, USA), anti-CXCR4 polyclonal antibody (35-8800, Invitrogen, Waltham, MA, USA ), anti-TSG101 monoclonal antibody (4A10, Abcam), anti-human CD63 polyclonal antibody (556019, BD Biosciences) and anti-cytochrome C antibody (10993-1-AP, Proteintech). The BioRad ChemiDoc Touch imaging system (BioRad, Hercules, CA, USA) was used to analyze the bands after incubation with the corresponding goat anti-mouse- or goat anti-rabbit-HRP secondary antibody conjugates (1:5000 dilutions). The densitometry readings of each band were calculated by ImagJ (NIH, Bethesda, MD, USA).

#### 4.2.5. Transmission Electron Microscopy (TEM)

Morphology of the isolated NKEXOs was studied by TEM. A small drop (about 10 μL) of exosomes was dropped onto the Formvar/carbon-coated TEM grid. After settling for approximately 1 min, excess water was removed by touching the grid with a piece of filter paper. Next, the grid was covered with a small drop of 2% uranyl acetate. After drying for ten seconds, the copper nets were examined with a Tecnai T10 TEM (FEI, Oregon, OH, USA) operated at 120 kV. The morphology of the PAMAM dendrimers and NNs was also imaged by TEM following similar steps.

#### 4.2.6. Samples Labeling

To label isolated exosomes and let-7a loaded PAMAM, different labeling assays were performed. We added 2 μL of red lipophilic fluorescent dye PKH26 (Sigma-Aldrich, St. Louis, MO, USA) to 500 μL of Diluent C (Sigma-Aldrich). The mixture was then added to the isolated NK derived exosomes and mixed gently for 5 min at room temperature followed by addition of 1% BSA (1 mL) to facilitate the binding of the excess dye. Next, samples were washed with PBS and ultracentrifuged at 100,000 g for 70 min and the pellets resuspended in PBS. The fluorescent dye 1,10-dioctadecyl-3,3,30,30-tetramethylindotricarbocyanine iodide (DiR; Invitrogen) was also used to label exosomes. Purified exosomes were incubated in 1 μM DiR at a concentration of approximately 350 μg of exosomes per mL for 15 min at 37 °C, and washed as described above. FAM-labeled miRNA and cy5-labeled miRNA were both synthesized by Genepharma (Shanghai, China).

### 4.3. Cellular Uptake Assay

PKH26-labeled NKEXOs were incubated with MDA-MB-231 cells for 24 h in a humidified 5% CO_2_ atmosphere at 37 °C. Subsequently, cells were washed twice with PBS and fixed with 4% paraformaldehyde for 20 min. Aliquots (5 μL) of the cell suspension were placed in clear microscope slides, covered with a drop of FluorSave^TM^ Reagent (Invitrogen) and mounted with a coverslip. Images, including z-axis projection images, were taken using a Nikon A1R (Nikon instruments, Melville, NY, USA) confocal laser scanning microscope. The uptake of the NN/NKEXO cocktail by CHLA-255 cells was also imaged following similar steps after incubating for 12 and 24 h.

### 4.4. Flow Cytometry

FAM-labeled let-7a loaded PAMAM and PKH26-labeled NKEXOs were coupled to fabricate the nanoparticle cocktail as indicated before. To quantify the cellular uptake of the cocktail by tumor cells, CHLA-255 cells (10^5^ cells per well) were cultured with the NN/NKEXO cocktail made of let-7a (0.5 µg) and NKEXOs (50 µg), PBS or equivalent 293N/293Exo cocktail between 12 to 48 h, harvested, and washed twice with PBS. Then, the samples were centrifuged and the supernatants decanted. Cells were resuspended and collected before analysis with a Cytoflex flow cytometer (Beckman Coulter, Life Science, Indianapolis, IN, USA).

### 4.5. Preparation of the NN/NKEXO Cocktail

To prepare NN/NKEXO cocktails in this study, 0.05/0.5/5.28 μg let-7a mimics (Genepharma) were added to dendrimers following manufacturer’s instructions (SL100568, SignaGen Laboratories, Rockville, MD, USA) and mixed thoroughly to react at 4 °C for 15 min. The resulting let-7a loaded PAMAM were precipitated at high-speed (10,000 rpm) for 30 min to remove excess let-7a. Subsequently, 40/50/100 μg of NKEXOs suspension were added to the precipitated let-7a loaded PAMAM respectively and the mixture incubated for 24 hours at 4 °C. To obtain NNs, the NN/NKEXO cocktail was precipitated at high-speed (10,000 rpm) for 30 min and resuspended in PBS. The supernatants with excess NKEXOs were used for TEM and the precipitated NNs for both Western blotting analysis and TEM. The same procedure was used in fabricating another cocktail in this study.

Confocal laser scanning microscopy (Nikon A1R) was used to validate whether NKEXOs were successfully fused with the let-7a loaded-PMAM. Exosomes and let-7a mimics were fluorescently labeled by PKH26 and FAM, respectively. Laser excitation of PKH67 and FAM was done sequentially using 586- and 488-nm lasers.

### 4.6. In Vitro Cytotoxicity

To evaluate NKEXO cytotoxicity, MDA-MB-231 breast cancer cells and CHLA-255 neuroblastoma cells were transfected with the firefly luciferase gene to quantify target cell survival using bioluminescence. MDA-MB-231-luc and CHLA-255-luc cells (10^4^ cells per well) were cultured with different quantities of NKEXOs (10, 20 and 40 μg) in clear-bottomed 96-well plates for 24 h. The culture medium was discarded and cells washed with PBS three times, followed by the addition of 120 μL of cell lysis buffer from a luciferase reporter Gene Assay kit (Yisheng, Shanghai, China). The 96-well plates were then shaken in a micro-shaker at room temperature for 15 min to fully lyse the cells. Next, the pyrolyzed lysates were centrifuged at 10,000 rpm for 3–5 minutes. After centrifugation, 100 μL of the supernatants from each well were transferred to a clear-bottomed 96-well plate, and 100 μL of firefly luciferase assay reagent added. Bioluminescent signals of the mixture were measured by the IVIS^®^ Lumina III imaging system (PerkinElmer, Santa Clara, CA, USA) and expressed as photon flux (photons/s). All experiments were done in triplicate from independent cell cultures.

To evaluate the cytotoxicity of cocktail-let-7a against the neuroblastoma cells in vitro, CHLA-255-luc cells (10^4^ cells per well) were treated with NKEXOs, let-7a alone or cocktail-let-7a containing 40 μg NKEXOs and 0.05 μg let-7a. Bioluminescent signals of the mixture were measured by an automatic ELISA plate reader and the relative light unit read at 562 nm.

### 4.7. In Vivo Animal Experiments

In vivo analysis was performed using specific pathogen-free, 6-week-old, female NOD/SCID mice (Slaccas, Shanghai, China). All animal experimental protocols were conducted in accordance with all national guidelines and regulations, and approved by the Animal Ethics Committee (NO. 2018R03042).

#### 4.7.1. In Vivo Targeting Capability of the Nanoparticle Cocktail

The cocktail was labeled with Cy5-let-7a loaded PAMAM or DiR-NKEXOs. Then, 5 × 10^6^ CHLA255 cells were intravenously injected into mice, which were randomly separated into four groups after 21 days in captivity. Animals in one group received intravenous injections containing 400 µL of labeled cocktail with identical amounts of let-7a (5.28 µg) and NKEXOs (100 µg). A second group received let-7a loaded PAMAM and the third group received let-7a only. At the end of the experiment (6 h after injection), the mice were euthanized and dissected tissues (heart, lung, liver, kidneys, spleen, livers and tumors) were imaged immediately. Fluorescence imaging was performed using the IVIS^®^ Lumina III imaging system. Background fluorescence was measured and subtracted by setting up a background measurement at time of data acquisition. A separate in vivo biodistribution study was performed using the following procedure. CHLA-255-luc cells (2 × 10^7^ cells/200 μL) were subcutaneously injected into the forward thighs of NOD/SCID mice. After 14 days, the mice were anesthetized and the subcutaneous tumor tissue was carefully exposed using surgical scissors. Mice were injected intravenously with 400 μL of a cocktail made of FAM-labeled miRNA let7a (5.28 µg) and PKH26-labeled NKEXOs (100 μg). After 20 min, in vivo two-photon confocal microscopy (Olympus BX61, Olympus America, Center Valley, PA, USA) was performed to detect each fluorescence signal from the subcutaneous tumor.

#### 4.7.2. In Vivo Antitumor Assay

The ability of the cocktail to inhibit tumor growth was evaluated in a neuroblastoma tumor-bearing mouse model. Fifteen mice were used in this experiment. Briefly, CHLA-255-luc cells (10^7^ cells/500 μL) were i.v. injected into the NOD/SCID mice. Mice were randomly separated into five groups (*n* = 3) 14 days later, and intravenously injected with different cocktail formulations: NKEXOs, cocktail-let-7a, PAMAM, let7a or with PBS as control group at let-7a dose of 5.28 µg and NKEXO dose of 100 µg. The same treatments were repeated after three days. After treatment, bioluminescence imaging (BLI) was performed with the IVIS^®^ Lumina III imaging system to analyze the cocktail’s therapeutic effect.

### 4.8. Statistics

Data are expressed as mean ± SD. SPSS Statistics 24 software (IBM, Armonk, NY, USA) and GraphPad Prism 5 software (GraphPad Software, Inc., San Diego, CA, USA) were used to perform data analysis using the analysis of variance (ANOVA) function. *p* < 0.05 values were considered statistically significant.

## 5. Conclusions

**Figure 5:**
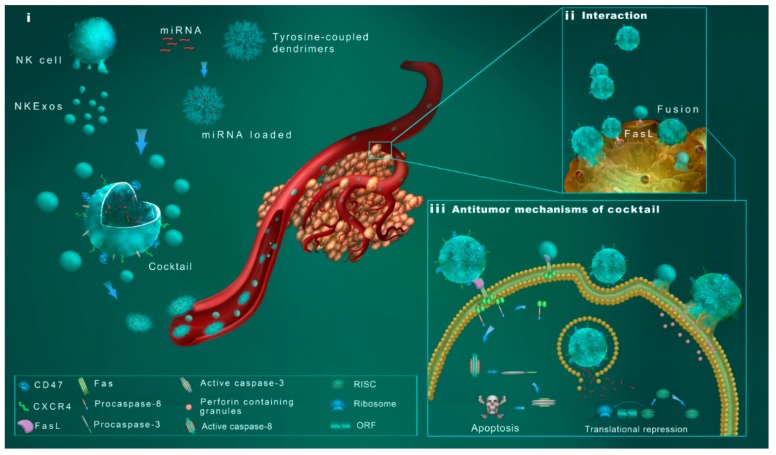
Schematic design of the NN/NKEXO cocktail for tumor targeting and drug delivery. (**i**) Main components of the cocktail: exosomes derived from NK cells (NKEXOs); exosome camouflaged core–shell nanoparticles (NNs). CD47 and CXCR4 facilitate a more accurate and active delivery of NNs. (**ii**) Interaction of the nanoparticle cocktail with target cells. Both NKEXOs and NNs are involved in exosome-to-membrane fusion and receptor-ligand interaction. (**iii**) Mechanisms of cocktail antitumor effect include perforin/granzyme mediated apoptosis, FasL/Fas mediated apoptosis and the effect of released therapeutic miRNA.

In summary, we reported the novel strategy for tumor targeting therapy based on NN/NKEXO cocktail. As displayed in Figure 5, the NNs constituted a novel biomimetic core–shell polymer, formed by the self-assembly of two components: (1) A PAMAM dendrimer-based inner core part for loading gene therapeutic agents and (2) the NKEXO based outer shell facilitating tumor targeting and acting as a direct antitumor agent. The NKEXOs coating guided NNs to the tumor and interacted with the plasma membrane of the target cells via endocytosis/fusion or via FasL/Fas. Finally, the therapeutic miRNA was released and regulated the transcriptome batch or gene of the cell, which performed a combined anti-tumoral effect with NKEXOs.

## Supplementary Materials

The following are available online at https://www.mdpi.com/2072-6694/11/10/1560/s1, Figure S1: Confocal laser scanning microscopy examination of MDA-MB-231 cellular uptake of NKEXOs; Table S1: Parameters of high-content screening (HCS) analysis; Video S1: A rotating 3D rendering of z-stack images showing the internalization of NKEXOs into MDA-MB-231-luc cells; Video S2: Fluorescence signal of NN/NKEXO cocktail detected in the tumor tissues using TPEFI.
