# Supplementary material for: Cocktail Strategy Based on NK Cell-Derived Exosomes and Their Biomimetic Nanoparticles for Dual Tumor Therapy
Source: Cancers (Basel). 2019 Oct 14;11(10):1560. doi: 10.3390/cancers11101560 (PMC6827005; doi:10.3390/cancers11101560)
Supplement: Supplementary file 1 [file cancers-11-01560-s001.zip › cancers-609450 supplementary after proof/cancers-609450-supplementary-after proof.pdf]

Supplementary Materials

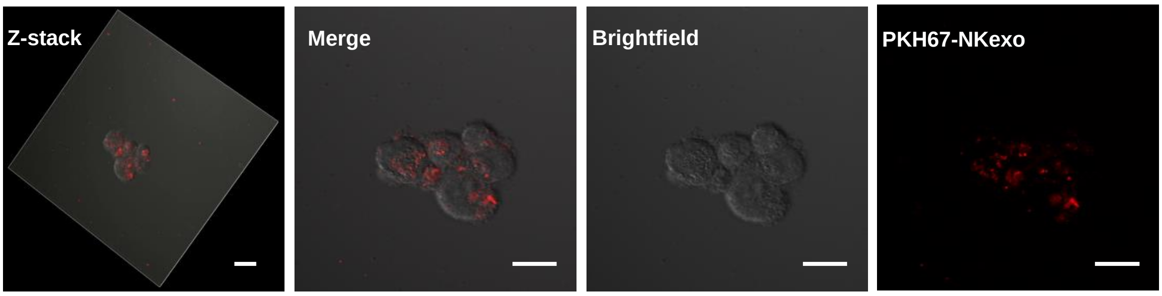

Figure S1. Confocal laser scanning microscopy examination of MDA-MB-231 cellular uptake of NKEXOs. Fluorescence images were taken after 24 h incubation with NKEXOs.

Table S1. Parameters of high-content screening (HCS) analysis.

**1. Input Image**	**6. Select Population (2)**	**11. Calculate Intensity Properties**
Stack Processing: Individual Planes	Population:488 Selected	Channel: Alexa568
Flatfield Correction: Basic	Method: Filter by Property	Population: 568 Selected
	Intensity 488 Mean > 550*	Region: Image Region Alexa568
**2. Find Image Region**	Output population: 488 Selected	Method: Standard (Mean)
Channel: Alexa488		Output Properties: Intensity 568 Selected
ROI: None	**7. Calculate Intensity Properties**	
Method: Common Threshold (0.47*)	Channel: Alexa488	**12. Calculate Intensity Properties**
Output population: Image Region 488	Population: 488 Selected	Channel: Alexa488
Output Region: Image Region 488	Region: Image Region 488	Population:568 Selected
	Method: Standard (Mean)	Region: Image Region Alexa568
**3. Calculate Morphology properties**	Output Properties: Intensity 488 Selected	Method: Standard (Mean)
Population: Image Region 488		Output Properties: Intensity 488 in 568
Region: Image Region 488	**8. Find Image Region**	
Method: Standard	Channel: Alexa568	**13. Select Population**
Area: μm^2	ROI: None	Population:568 Selected
Output Properties: Image Region 488	Method: Common Threshold (0.34*)	Method: Filter by Property
	Output population: Image Region 568	Intensity 488 in 568 > 550*
**4. Select Population**	Output Region: Image Region 568	Output population: merge
Population: Image Region 488		
Method: Filter by Property	**9. Calculate Morphology properties**	**14. Define Results**
Image Region 488 Area (μm^2) < 20*	Population: Alexa568	Method: List of Outputs
Output population: 488 Selected	Region: Image Region Alexa568	Method: Formula Output
	Method: Standard	Formula: a/b
**5. Calculate Intensity Properties**	Area: μm^2	Variable A: merge-Number of Objects
Channel: Alexa488	Output Properties: Image Region Alexa568	Variable B:568 Selected-Number
Population:488 Selected		
Region: Image Region 488	**10. Select Population**	
Method: Standard: Mean	Population: Alexa568	
Output Properties:Intensity 488	Method: Filter by Property	
	Image Region Alexa568 Area (μm2) <30*	
	Output population: 568 Selected	

* indicate μm2.

Supplementary Videos

Video S1. A rotating 3D rendering of z-stack images showing the internalization of NKEXOs into MDA-MB-231-luc cells.

Video S2. Fluorescence signal of NN/NKEXO cocktail detected in the tumor tissues using TPEFI.
