# Supplementary material for: Cocktail Strategy Based on NK Cell-Derived Exosomes and Their Biomimetic Nanoparticles for Dual Tumor Therapy
Source: Cancers (Basel). 2019 Oct 14;11(10):1560. doi: 10.3390/cancers11101560 (PMC6827005; doi:10.3390/cancers11101560)
Supplement: Supplementary file 1 [file cancers-11-01560-s001.zip › cancers-609450 supplementary after proof/Supplemental Materials for Original WB pictures.pptx]

## Slide 1
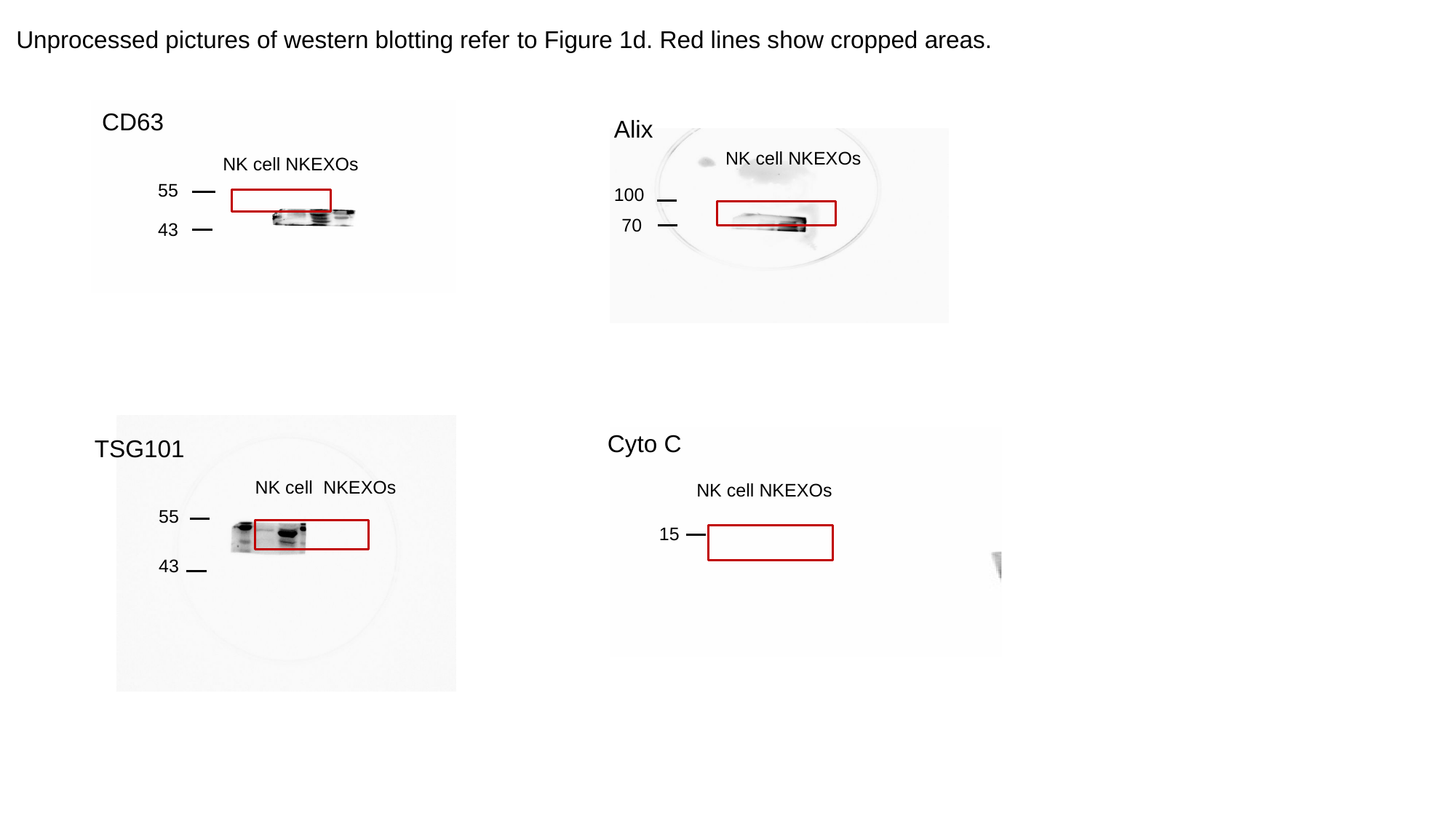

Unprocessed pictures of western blotting refer to Figure 1d. Red lines show cropped areas.
NK cell
NKEXOs
55
43
CD63
Alix
NK cell
NKEXOs
100
70
TSG101
NK cell
NKEXOs
55
43
Cyto C
NK cell
NKEXOs
15

## Slide 2
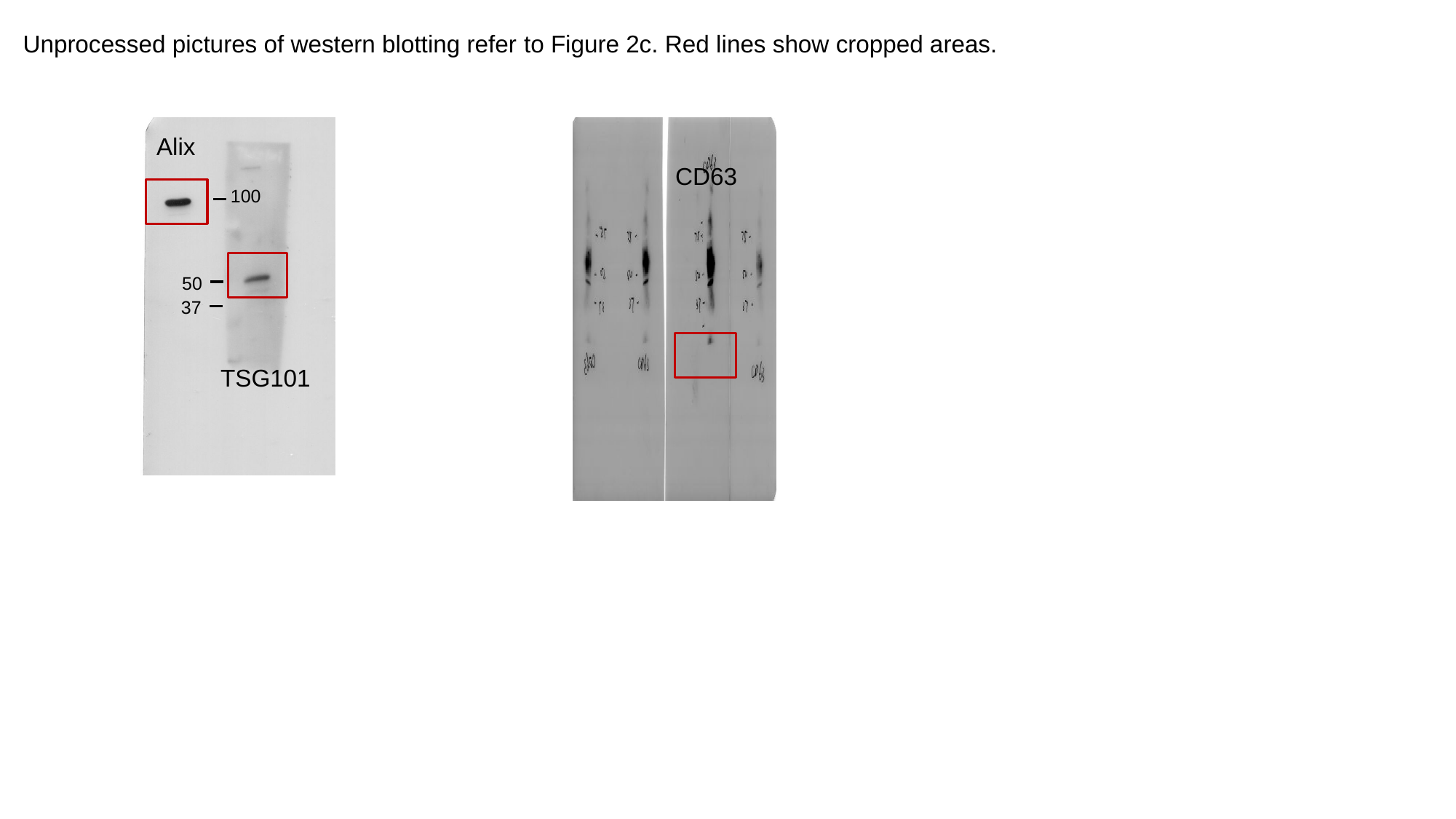

Unprocessed pictures of western blotting refer to Figure 2c. Red lines show cropped areas.
Alix
100
50
37
TSG101
CD63

## Slide 3
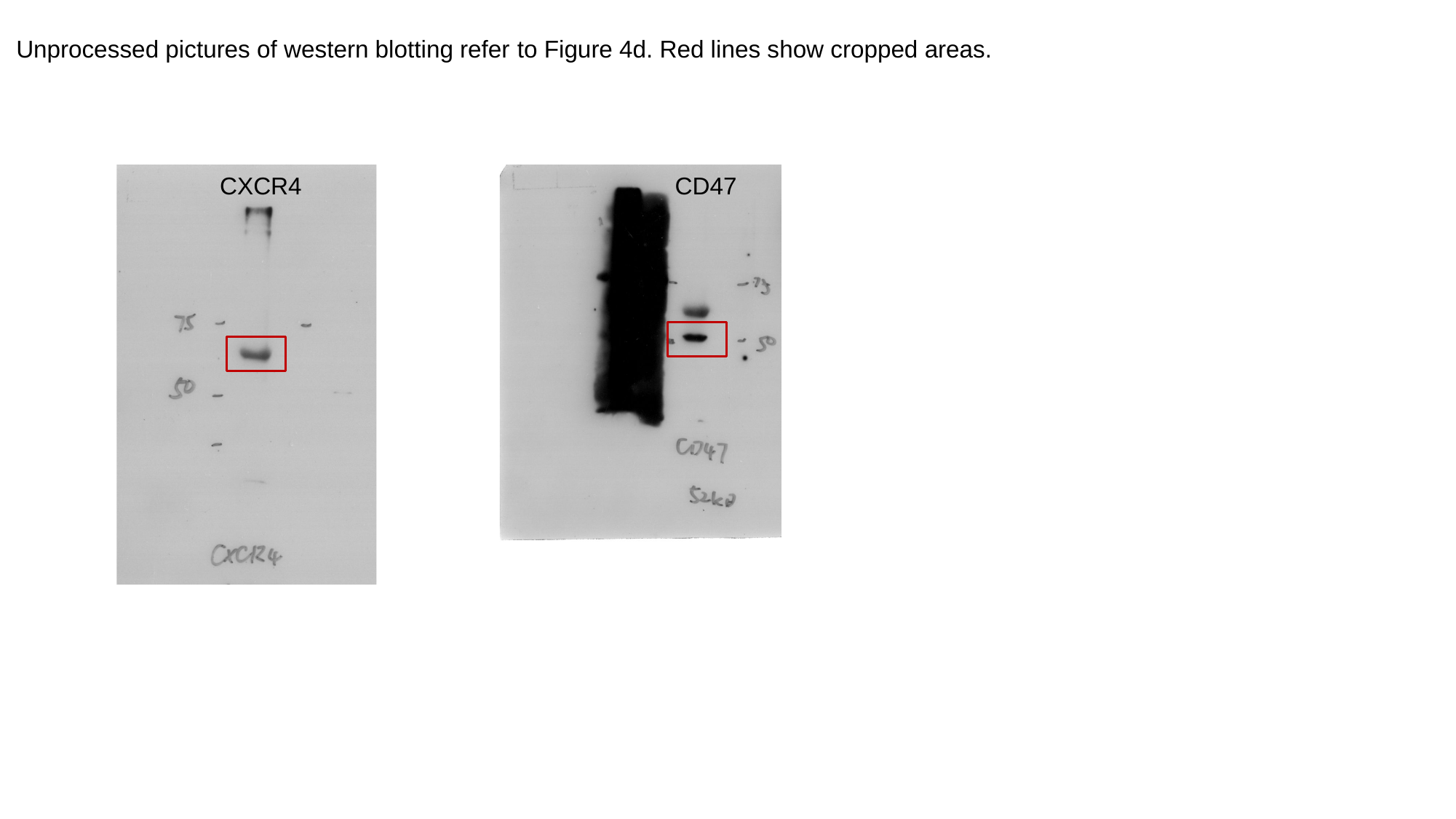

Unprocessed pictures of western blotting refer to Figure 4d. Red lines show cropped areas.
CXCR4
CD47
